# Verification of the accuracy of 3D calculations of breast dose during tangential irradiation: measurements in a breast phantom

**DOI:** 10.1120/jacmp.v2i3.2608

**Published:** 2001-09-01

**Authors:** Steven M. Kirsner, Karl L. Prado, Ramesh C. Tailor, José A. Bencomo

**Affiliations:** ^1^ Department of Radiation Physics The University of Texas, M. D. Anderson Cancer Center 1515 Holcombe Boulevard Houston Texas 77030

**Keywords:** breast tangent irradiation, breast phantom, treatment‐planning system verification, monitor units

## Abstract

This report specifically describes the use of a unique anthropomorphic breast phantom to validate the accuracy of three‐dimensional dose calculations performed by a commercial treatment‐planning system for intact‐breast tangential irradiation. The accuracy of monitor‐unit calculations has been corroborated using ionization chamber measurements made in this phantom. Measured doses have been compared to those calculated from a variety of treatment plans. The treatment plans utilized a 6‐MV x‐ray beam and incorporated a variety of field configurations and wedge combinations. Dose measurements at several clinically relevant points within the breast phantom have confirmed the accuracy of calculated doses generated from the variety of treatment plans. Overall agreement between measurements and calculations averaged 0.998±0.009. These results indicate that the dose per monitor‐unit calculations performed by the treatment‐planning system can be confidently utilized in the fulfillment of clinical dose prescriptions.

PACS number(s): 87.53.–j, 87.66.–a

## I. INTRODUCTION

Treatment‐planning systems routinely utilize beam data obtained through measurements made in a water phantom. During commissioning of a treatment‐planning system, calculations from the system are verified against either measurements or “hand calculations” performed for a series of configurations designed to represent clinical situations.^1^ ^,^ ^2^ Invariably, these configurations utilize a box‐like water phantom geometry similar to the geometry used for beam data acquisition. Hand calculations also assume this same geometry. Thus, planning systems are tested and found to be accurate under conditions that mimic a water phantom.

Since current treatment‐planning algorithms now explicitly account for the 3‐dimensional (3D) volume of irradiated tissue, it is necessary to verify dose calculations performed in geometries significantly different than that of a water phantom. One such geometry is that of the intact breast irradiated tangentially. Leszczynski and Dunscombe^3^ verified the accuracy of dose calculations performed on a commercially available 3D treatment‐planning system (Helax‐TMS; Helax AB, Upsala, Sweden) by comparing the planning system's results with manual calculations. Loshek *et al.*,^4^ verified the accuracy of 3D dose calculations performed on a second commercial planning system (ADAC Pinnacle;^3^ ADAC Laboratories, Milpitas, CA) by comparing this planning system's results with measurements made in a solid water phantom. Although these reports conclude that 3D calculations properly account for scatter in most circumstances, they fail to specifically address the complex geometry of the intact breast. Most recently, Baird *et al.*,^5^ compared ADAC Pinnacle^3^ calculations with measurements in a solid wax breast phantom. They used radiographic film and thermoluminescent dosimeters to measure dose distributions in a sagittal plane at breast mid‐separation.

The verification of dose calculations in 3D treatment planning of the breast has particular importance because tangential breast irradiation presents several challenging dosimetric conditions. The volume of irradiated breast is significantly smaller than most other clinical volumes. Dose normalization points are often located either close to beam edges or to tissue boundaries, and beams are often obliquely incident. Corrections for tangential‐field “flash” or “fall off” do not fully account for the total reduction in volume.

In this work, the accuracy of absolute homogenous dose calculations performed by the ADAC Pinnacle^3^ treatment‐planning system for intact breast tangential irradiation was evaluated using an anthropomorphic breast phantom designed specifically for this purpose. The phantom was scanned by computed tomography (CT) and 3D planned. Ionization‐chamber measurements were then made at several clinically relevant positions within the phantom under different irradiation conditions. The dose measurements were then compared with dose calculations to evaluate the accuracy of the calculation.

## II. MATERIALS AND METHODS

### A. Breast phantom description

**Figure 1:**
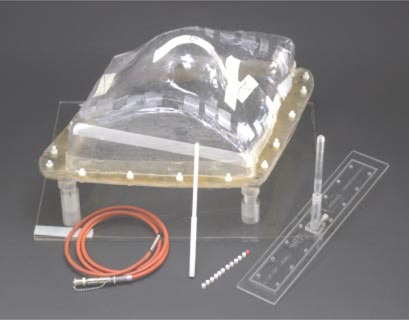
(Color) The anthropomorphic breast phantom, ion‐chamber sleeve, and chamber localization device and spacers are shown, as well as the waterproof PTW ion chamber used for measurements. The superior and lateral portions of the phantom are shown on the top and right sides of the figure, respectively.

Measurements of ionization were made in an anthropomorphic breast water phantom (Fig. 1) that was originally designed by the Radiological Physics Center (RPC) and a collaborator (D. A. Viggars, Cancer Care Manitoba, Winnipeg, Manitoba, Canada), to be a transportable device used to monitor institutions participating in national protocols. An important consideration in the design of the phantom was the reproduction of a geometry that could realistically duplicate the female torso in treatment position. With this in mind, an impression of the entire thorax of a patient was obtained. This impression was used to create a thermoplastic shell of the patient's left thorax. The shell encompassed an area extending from the left shoulder superiorly, to approximately mid‐abdomen inferiorly, beyond the sternum medially, and past the edge of the chest laterally. In each step of the process, faithful transfer of patient laser, field border, and central axis markings, obtained during patient simulation, was achieved. The superior and inferior edges of the shell were squared off, and the resulting mold was then fitted with a watertight flat acrylic (polymethylmethacrylate) backing. A valve was incorporated within the acrylic backing to allow the phantom to be filled with water and later drained. Four pegs are fixed onto a plastic base to support the main body of the breast phantom.

**Figure 2:**
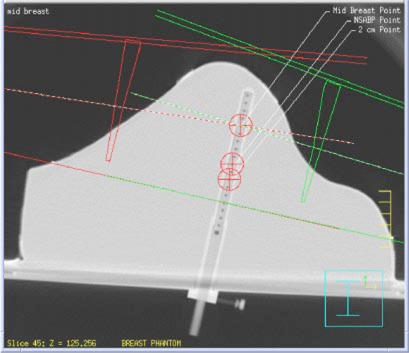
(Color) CT slice of the anthropomorphic breast phantom showing the ionization chamber insert and polystyrene localization assembly, and the three points (mid‐breast, NSABP, and 2‐cm) used for both isocentric set‐up and dose calculation. The isocenter of the fields shown is at mid‐breast.

An acrylic ion chamber sleeve, as also shown in Fig. 1, allows insertion of a 0.125 cm^3^ waterproof ion chamber. The sleeve was constructed by machining an acrylic tube to make its interior dimensions match the exterior dimensions of a Physikalisch‐Technische Werkstatatten (PTW) Model N233642 ion chamber (PTW‐New York, Hicksville, NY). The wall of the sleeve was 2.7 mm thick. The sleeve is mounted onto a base that can then be attached to the flat back of the phantom. This permits the ion chamber to be inserted from below. The assembly has been designed to allow the ion chamber to be placed at any point along a perpendicular bisector located at the midpoint of the line connecting the posterior borders of the medial and lateral tangential fields. The position of the chamber along the bisecting line is localized using a polystyrene rod with dimensions equal to those of the ion chamber. Position markers, consisting of 1 mm diameter holes drilled through the localization rod, are located along the length of the rod in 7 mm (the length of the chamber's sensitive volume) increments. The chamber‐localization device allows high‐contrast visualization, during CT imaging of the phantom, of possible chamber measurement positions (Fig. 2). Prior to irradiation of the phantom, positioning of the chamber at a point of interest was achieved by inserting an appropriate number of polystyrene spacers in the sleeve before inserting the ion chamber. Spacers replace the air cavities in the irradiated volume.

### B. Breast phantom simulation/CT simulation

The medial, lateral, superior, and inferior field borders previously marked on the phantom, were delineated with chrome wire (0.38 mm in diameter). For CT data acquisition, the phantom, with chamber positioning device, was placed in treatment position and a full scan was performed. Three treatment isocenters were chosen and localized along the perpendicular bisector of the line between the posterior edges of the medial and lateral tangential fields. A first point was centrally located within the impression of the breast. A second point corresponded to the National Surgical Adjuvant Breast and Bowel Project (NSABP) calculation point: a point located two‐thirds of the distance from the apex of the breast to the posterior edge of the tangential fields. A third point was located at a nominal 2.0 cm distance from the posterior field edge. The first two isocenters are believed to be representative of those that are commonly used clinically (mid‐breast and NSABP); the third was placed near the field edge to test an extreme case that is not uncommonly encountered. The location of these points is shown in Fig. 2.

The phantom underwent “virtual simulation” for each isocenter position using the proposed field borders marked on the breast phantom. In all cases, gantry angles were set such that the divergence of each beam matched posteriorly. Collimator angles were determined using the field border wires to match the field edge to the wire. The field sizes were chosen to match the field borders and to give approximately 2 cm of fall off or flash beyond the breast tissue.

### C. Breast phantom treatment planning

Treatment plans using 6 MV beams were developed for each isocenter position using the ADAC Pinnacle^3^ treatment‐planning system (Table I). Plans were optimized to achieve the most uniform dose distribution given the field borders that had been initially simulated by the RPC collaborators and the wedge angles selected. This optimization was achieved by varying the beams’ weighting, yet limiting the irradiation to divergence‐matched opposed tangential fields. As an additional consideration, the treatment plans were devised trying to use as many possible wedge combinations that could be used in a clinical environment, with the exception of Plan 4, which intentionally used no wedges. A total of four treatment plans were developed using the isocenter positions virtually simulated.

**Table I:** Breast‐phantom treatment plans showing isocenter locations and treatment‐field specifics. Asymmetric collimator settings are identified by using a colon (:) between each asymmetric $\text{jaw}-X_{1}:X_{2}\times Y$.

Plan	Isocenter	Field	Gantry	Coll.	Field Size	Wedge	SSD	
1	mid‐breast	medial	279	271	7.0:7.0*22.0	15	91.5
		lateral	107	89	7.0:7.0*22.0	15	95.2
2	mid‐breast	medial	279	271	7.0:7.0*22.0	none	91.5
		lateral	107	89	7.0:7.0*22.0	30	95.2
3	NSABP	medial	281	272	10.0:3.0*22.0	15	88.5
		lateral	105	88	10.0:3.0*22.0	45	87.0
4	2‐cm	medial	282	271	12.0:1.8*22.0	none	87.1
		lateral	104	89	12.0:1.8*22.0	none	86.0

In each plan, 200 cGy was delivered to isocenter. The contribution of each field to the total dose at each measurement point is presented in Table II. Dose was calculated using the Pinnacle^3^ Collapsed Cone Convolution^6^ homogenous dose engine. In each treatment plan, and for each field, dose was calculated to three points. The three points were the treatment isocenter and each of the other isocenter locations described earlier. Calculation to the other points was chosen to test the Pinnacle^3^ ability to calculate dose at off‐axis points along a wedged direction.

**Table II:** Description of dose calculation points, and results of calculations and measurements.

Field No.	Plan No.	Isocenter location	Calculation point	Treatment field	Calculated dose (cGy)	Measured dose (cGy)	Measured/calculated
1	1	mid‐breast	mid‐breast	medial	94.2	94.4	1.002
2			mid‐breast	lateral	105.9	105.9	1.000
3			NSABP	medial	90.4	89.8	0.993
4			NSABP	lateral	84.0	84.3	1.003
5			2‐cm	medial	86.0	84.1	0.978
6			2‐cm	lateral	81.3	80.0	0.983
7	2	mid‐breast	mid‐breast	medial	93.6	93.4	0.998
8			mid‐breast	lateral	106.2	107.6	1.014
9			NSABP	medial	86.2	85.8	0.995
10			NSABP	lateral	89.0	89.9	1.010
11			2‐cm	medial	80.8	79.5	0.983
12			2‐cm	lateral	87.4	86.4	0.989
13	3	NSABP	mid‐breast	medial	115	115.4	1.004
14			mid‐breast	lateral	109.8	110.3	1.005
15			NSABP	medial	104.3	105.0	1.006
16			NSABP	lateral	96.1	97.5	1.014
17			2‐cm	medial	99.7	98.5	0.988
18			2‐cm	lateral	97.8	97.7	0.999
19	4	2‐cm	mid‐breast	medial	126.5	125.6	0.993
20			mid‐breast	lateral	149.9	149.0	0.994
21			NSABP	medial	108.4	107.9	0.996
22			NSABP	lateral	106.3	106.6	1.003
23			2‐cm	medial	99.9	99.7	0.998
24			2‐cm	lateral	99.7	98.8	0.991

### D. Breast phantom irradiation

The phantom and ion chamber assembly were irradiated utilizing the 6‐MV x‐ray beam of a Siemens Mevatron 6740 MXE linear accelerator (Siemens Medical Systems‐OCS, Concord, CA). Ionization was measured with a PTW N, 233642 ionization chamber and a CNMC Model 206 electrometer (CNMC Company, Inc., Nashville, TN). Prior to phantom irradiation, the chamber/electrometer response per unit dose was determined by cross comparison using a $10\times 10\;{\text{cm}}^{2}$ field, 100 cm. Source to Surface distance (SSD), and a depth of $d_{\max}$ in water, with a system of known calibration. Chamber readings, during both calibration and experiment, were corrected for temperature and pressure, to account for possible differences between calibration and experiment conditions.

For each phantom irradiation, the chamber was placed at its first measurement position and the first isocenter was set. SSD's were verified for both medial and lateral fields. The first set of tangential fields were then treated using the monitor units calculated by the Pinnacle.^3^ The phantom was then moved, as appropriate, to the next isocenter position where the next set of fields were verified and treated. The fields of all isocenters were irradiated before the chamber was repositioned and the process repeated. A total of 24 dose measurements were obtained over two evenings; select measurements were repeated to ascertain measurement precision.

## III. RESULTS

### Phantom measurements

Ionization measurements made over two evenings demonstrated a precision better than 0.2% (coefficient of variation).^7^ A total of 24 dose measurements were compared with their respective dose calculations. Table II shows the results of the comparison between calculations and measured data. As seen in the table, the ratio of measurement to calculation for each separate field ranges from 0.978 to 1.014. The mean of the 24 ratios was 0.998; the standard deviation of the group was 0.009 (0.9%).

**Figure 3:**
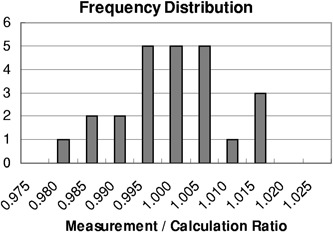
Frequency distribution of measurement to calculation ratios.

The distribution of the measured to calculated ratios can be seen in the histogram of Fig. 3. The data appear to be equally distributed about 1.0, indicating that no systematic errors existed either in the calculation or in the set‐up and irradiation of the breast phantom. Of the 24 measurements, all but one agreed with calculations to within 2% (a commonly used criterion of dose‐calculation algorithm accuracy),^8^ with most (two‐thirds) agreeing within 1%.

## IV. DISCUSSION

**Figure 4:**
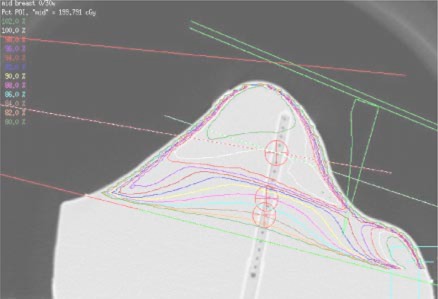
(Color) Isodose distribution produced in Plan 2. The isocenter of the fields is located at the mid‐breast point; the medial field is open and the lateral field has a 30‐degree wedge. Isodose lines range from 102% to 80% in 2% increments.

The dose‐calculation verification measurements performed in this study utilizing an anthropomorphic breast phantom clearly demonstrate absolute homogenous dose calculation accuracy – even in the case of tangential irradiation of the breast. This high degree of accuracy is achievable when a treatment‐planning system and its beam data are appropriately modeled and commissioned.^1^ ^,^ ^2^ The planning system's calculation algorithm was tested for a variety of standard breast irradiation techniques, and under multiple calculation conditions, such as off axis points, along wedged gradients, and at points close to the field edge. For the range of calculation conditions, the planning system was able to predict the dose accurately. The worst case resulted in an error of only 2.2%, with all other data points well within 2%. Two‐thirds of the measurements agreed with calculations to within 1%. The majority of cases where the differences between measurement and calculations exceeded 1%, occurred at the nominal 2‐cm point, where, because of proximity to field edges and lateral contour irregularity, dose gradients at the measurement point exist. This is shown in Fig. 4.

Our breast phantom results are in good agreement with previously published results for conventional phantoms^3^ ^,^ ^4^ and in excellent conformity with the results recently published for a breast phantom.^5^ Using film and (TLD), Baird *et al.*,^5^ evaluated the concurrence between calculations and measurements in a wax breast phantom. The dose distributions of 6 and 18 MV treatment plans that were optimized using open/wedge combinations and compensator filters, were verified in a sagittal plane midway between the medial and lateral fields. They demonstrated agreement between calculations and measurements to within 3%. Potential systematic errors associated with film measurements, coupled with the 0.6% quoted precision, possibly precluded obtaining better accuracy. Our results, however, substantiate theirs, but to a more clinically acceptable accuracy.

Whereas Baird *et al.* have evaluated uniform dose distributions, we have evaluated both uniform dose distributions, as well as fields producing dose gradients within the irradiated volume.

In their report, Loshek *et al.*,^4^ have shown agreement better than 1% between calculations performed and measurements made in a solid water phantom, when irradiated with fields having varying percentages of flash or fall‐off. In that situation, however, the beam incidence was normal to the phantom surface. They have similar results for oblique incidence, but the two effects were not combined. In addition, their measurements were made in a phantom larger than most intact breasts. Leszczynski and Dunscombe,^3^ using clinical data, compared 3D computer calculations to manual calculations. Their results, which included breast/chest wall data, also show conformity well within 1%, although this was between computer‐generated and manual calculations, not with measured data. Other investigators^4^ have not been as successful in substantiating computer results with manual calculations, particularly in the case of the breast. This has been our experience as well. It appears likely that, as a consequence of Dunscombe's Helax‐TMS system validation experience,^9^ their manual calculation methodologies may have been appropriately modified to agree with their proven computer‐generated results. Nevertheless, the accuracy of 3D computations does appear to have been properly validated and the results presented here substantiate that fact.

## V. CONCLUSION

Measurements of dose made in a fairly unique anthropomorphic breast phantom have demonstrated excellent agreement for 6‐MV x‐ray beams (0.998±0.009), with calculations performed under a variety of treatment field configurations. The accuracy of point homogeneous dose calculations along an axis perpendicular to the edges of divergence‐matched tangents at mid‐separation, produced by a 3D system during intact‐breast treatment planning, has been confirmed. These results, coupled with those of Baird *et al.*,^5^ lead us to conclude that the dose per monitor unit calculations performed using Pinnacle's collapsed cone convolution in homogeneous mode, may be confidently used to implement clinical dose prescriptions, under any treatment conditions for breast tangent irradiation. Also, this work confirms the utility of an anthropomorphic “water” breast phantom as a tool to assess accuracy of a treatment‐planning systems photon dose calculation.

## ACKNOWLEDGMENTS

The authors would like express their appreciation to Jens Boving and Mark Bushman of the Department of Radiation Physics Instrument Shop for their expert craftsmanship in producing a phantom of great utility and excellent quality. Without their dedication, this work would not have been possible.
